# Implementation and validation of the F^4^aT laboratory for flow in rough fractures

**DOI:** 10.1038/s41598-025-34648-2

**Published:** 2026-01-13

**Authors:** Carola M. Buness, Fabian Nitschke, Thomas Kohl

**Affiliations:** Geothermal Energy and Reservoir Technology, Institute of Applied Geosciences, Karlsruhe Institute of Technology, Karlsruhe, 76131 Germany

**Keywords:** Energy science and technology, Engineering, Solid Earth sciences

## Abstract

Accurate characterization of fracture hydraulics is crucial for optimizing subsurface systems, notably geothermal energy extraction where high flow rates are essential for efficient energy production. The precise transition from linear to nonlinear fracture hydraulics at already moderate flow rates is still undefined, due to the complexity of fracture roughness, where the influence of various roughness parameters and the comparability of individual rough fractures are still unclear. Here, we introduce the *Forced Fluid Fracture Flow and Transport Laboratory* (F^4^aT-Hydraulic Laboratory), a novel experimental laboratory designed to address this knowledge gap. It focuses on a comprehensive workflow encompassing high-resolution measurements of the rock surface roughness and the experimental investigation of fracture hydraulics at a large range of flow rates ($0.05 < Re < 100$). A unique feature of the F^4^aT-Hydraulic Laboratory is its ability to conduct systematic and stochastic investigations of roughness-hydraulic interactions through 3D printed fracture replicas with defined, statistically varied fracture roughness. In this study, we present the developed workflow in detail, provide benchmarking experiments against analytical solutions, and demonstrate the ability to measure roughness effects on the transition from linear to nonlinear hydraulic regimes at already moderate flow rates ($Re \approx 10$).

**Supplementary Information:**

The online version contains supplementary material available at 10.1038/s41598-025-34648-2.

## Introduction

The study of subsurface fluid dynamics is crucial across various research fields, including groundwater contamination^1,2^, nuclear waste repositories^3^, and geothermal energy production^4^. In all these applications, the role of fractures cannot be overstated, as they often act as primary conduits, dominating the overall subsurface flow behavior and transport of heat and solutes. This is particularly relevant for the development of Enhanced Geothermal Systems (EGS), which rely on fractured reservoir systems and offer great potential for sustainable energy supply. The efficiency of these reservoirs depends on a reliable quantification of their hydraulic behavior, whereby high flow rates and low pressures are desired. In EGS where the rock matrix is virtually tight, flow is concentrated almost completely in fractures^4^, causing significantly higher local flow rates than in porous reservoirs. These locally high flow rates in fractures lead to high Reynolds numbers $Re$, potentially resulting in disproportionately high pressure gradients referred to as nonlinear hydraulic behavior. Field flow tests^5,6^ in specific fractured settings have found significant deviations from linear hydraulic reservoir response, where the flow rate is proportional to the pressure, thereby demonstrating the occurrence of nonlinear hydraulic behavior at field-scale.

For detailed reservoir analysis, reservoir development, and targeted reservoir management, reservoir engineering relies on numerical simulations of varying complexity, including coupled systems such as thermo-hydro-mechanical-chemical (THMC) models to simulate flow and transport phenomena in fracture networks. Depending on the objective, required problem complexity and domain size, different hydraulic models are established. To minimize the computational cost for hydraulic calculations of large-scale reservoir simulations, the simplified Cubic Law (CL) is often used. However, this linear analytic solution based on laminar flow between parallel plates is idealized and neglects the complex geometry and nonlinear high flow rate hydraulics in EGS. Addressing this, refinements have been made to the CL, incorporating fracture roughness, leading to various versions of modified or local CLs^7^. In contrast to the CL, which is parametrized with mean aperture values, pressure drop and flow rate across the entire domain scale, these localized laws account for small-scale fracture aperture variations and incorporate hydraulically effective aperture definitions. This enables the resolution of the local hydrodynamics for a known or measured fracture geometry. The large-scale Forchheimer Eq. ^8–10^ includes the average roughness by means of two coefficients and captures nonlinear hydraulic behavior by an additional quadric flow rate term. However, the coefficients for the roughness are predominantly derived from fitting to specific experimental data, thus representing a particular single fracture or fracture network and are not generally applicable. All details of fracture flow, such as channeling phenomena or local eddies/turbulence, which increase pressure losses, can be numerically simulated with the Navier–Stokes Equations by including the rough fracture surface details. These calculations provide precise local information on flow and pressure within the fracture, but they remain computationally highly expensive. This makes combining detailed fracture flow with large-scale reservoir domains prohibitive and already leads to restrictions in laboratory-scale models such as a reduced grid resolution^11^ or dimensional reduction^12^.

**Fig. 1:**
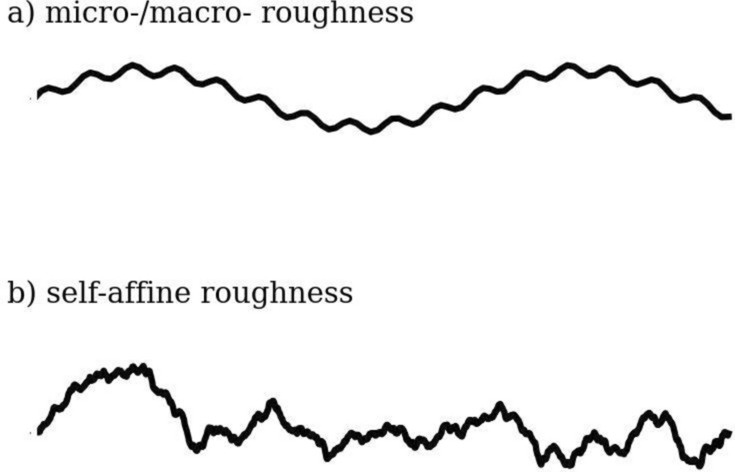
Different roughness model example profiles. The model of micro-/macro roughness with a roughness at just two frequencies a) and an example of a self-affine rough line where the roughness spans over the full frequency range.

The accuracy of all hydraulic and fluid dynamic simulations heavily relies on the roughness descriptions. However, the choice of the roughness quantification model still introduces an unclear degree of uncertainty into these simulations. It remains poorly understood how different models can be compared and to what extent the chosen roughness description influences fracture hydraulics. Two examples of the most commonly used models are the concept of micro-/macro roughness, also referred to as primary and secondary roughness (Fig. 1a), and the self-affine description, related to a fractal approach (Fig. 1b). The micro-/macro roughness concept assumes the roughness consists of a superposition of distinct large- and small-scale wavinesses^13,14^. In contrast, it is shown that most rock fracture roughness spans over all frequencies and exhibits a self-affine nature^15,16^, which leads to strongly sample size dependent statistics^17^. To statistically describe the roughness, the Hurst exponent $H$ is frequently used^15,16,18^, which is directly proportional to the slope of the roughness power spectrum. However, it lacks the roughness amplitude information, which leads to the necessity for at least a second parameter for the full roughness description. This is often the height standard deviation^19^$\sigma$ or the prefactor^16^$\widetilde{c}$from the calculation of $H$ by the use of the Power Spectrum.

In addition to the fracture surface roughness, the characterization of the fracture void, i.e. its complex 3D geometry, is crucial. The local void geometry strongly depends on the fracture model, whether normally opened joints or sheared faults are considered, where the combination of roughness and shear defines the local aperture. Therefore, the aperture $a$ can be defined in different ways. Here we define $a$ as the mechanical aperture, vertical to the overall fracture plane. However, using the effective aperture ${a}_{eff}$, which is vertical to the local flow direction, or a spherical aperture ${a}_{sphere}$, where locally maximized spheres define the aperture, could be more applicable for fracture flow dynamic analysis^20^. Taking the local aperture definitions into account, ${a}_{eff}$ and ${a}_{sphere}$ vary due to the locally different height gradients, even with constant $a$ and without shearing. These variations in aperture play a crucial role on the fracture flow, leading to flow channeling^11,20–24^, and increase when the fracture is sheared or includes closed contact areas. Shearing additionally introduces a directionality to the fracture void of initially isotropic rough surfaces , influencing the fracture flow^11^. Hydraulic and channeling experiments have been conducted by using fractured rocks^25–28^ or rock replicas^24,29–31^. While these experiments provide hydraulic data for the experiment-specific particular fracture rock sample, they lack the systematic investigation of key roughness parameters and their hydraulic sensitivity. Previous research by Louis^13^ dealt with the systematic investigation using (exposed aggregate) concrete fractures for micro-/macro roughness models, however, his findings can hardly be transferred to the self-affine model due to the lack of a defined characteristic roughness height for this model. Modern experimental techniques enable more precise fracture replica creations with 3D printing techniques, which permits the fabrication of designed self-affine surfaces. This methodology is employed to investigate the relationship between fracture geometry and the coefficients of the Forchheimer Eq. ^32^, to analyze the onset of the nonlinear hydraulic behavior in terms of $a$, roughness, and fracture networks^33^, and to measure flow paths and local velocities utilizing particle image velocimetry^34^. The innovative capability of fabricating rock replicas through 3D printing has resulted in a significant advancement in this field. Nevertheless, substantial research gaps persist at both laboratory-scale and field-scale due to the extensive diversity of fracture geometries. Notable examples include the hydraulic sensitivity to various roughness parameters independently, the definition of relative roughness for self-affine fractures, and the impact of distinct roughness parameters on channeling phenomena.

To complement these research efforts, the *Forced Fluid Fracture Flow and Transport Laboratory* (F^4^aT-Hydraulic Laboratory) was developed to provide the necessary control for systematic measurements. This facility allows for the comprehensive investigation of hydraulic sensitivity to different geometrical fracture parameters by combining fracture roughness analysis and hydraulic measurements of well-defined fracture geometries. The ability to use numerous fractures with statistically similar roughness enables a stochastic approach to account for natural roughness varieties, thus reproducing and validating numerical methods.

In this paper, we apply the methodology implemented in the F^4^aT-Hydraulic Laboratory to improve the understanding of fracture flow, starting from roughness measurements of rock fracture surfaces to hydraulic measurements of 3D printed fracture replicas. We first validate the flow-through test stand using parallel plate measurements against the linear CL theory, and we then demonstrate the complete workflow for rough granite surfaces exemplified by samples from the boreholes of the Soultz-sous-Forêts geothermal energy production site in France. The results highlight their hydraulic behavior differences compared to the parallel plate model.

## Methods

### Experimental requirements

Laboratory experiments focusing on fracture hydraulics applicable for geothermal energy production require setups capable of precise measurements of roughness, pressure and flow rate. In the following, the requirements for experiments with a 20 cm × 20 cm laboratory-scale fracture are presented considering $a \le 1$ mm and pure water as the working fluid. The aim is to experimentally investigate flow and transport phenomena in both linear and nonlinear hydraulic regimes, which requires Re between 0.1 and 100^9^. Most rocks, such as granite, exhibit significant fracture geometry variations, due to their heterogeneous mineral compositions and grain sizes, thereby impacting hydraulic properties. Consequently, the possibility of numerous measurements of comparable fractures is desirable to develop a generalized understanding of fracture geometry effects on flow, and to mitigate the uncertainty of single case-specific interpretations.

Variations in roughness can also be induced by alteration^35^ or different stress/strain conditions, with geometric and hydraulic implications for joints and faults according to their origin and development. To accurately capture roughness effects on hydraulics, multiple fractures need to be measured in order to statistically analyze roughness parameters and their distributions. This can be accomplished using techniques such as mechanical stylus profilometers^17^, LiDAR^16^ or various optical scanning methods like confocal profilometry. The scanning system must be capable of measuring an area up to the size of the fracture replica or sample, with a resolution several orders of magnitude smaller, ensuring a detailed representation of the fracture’s roughness. A roughness range spanning multiple orders of magnitude must be accomplished in the hydraulic measurements as well.

**Fig. 2:**
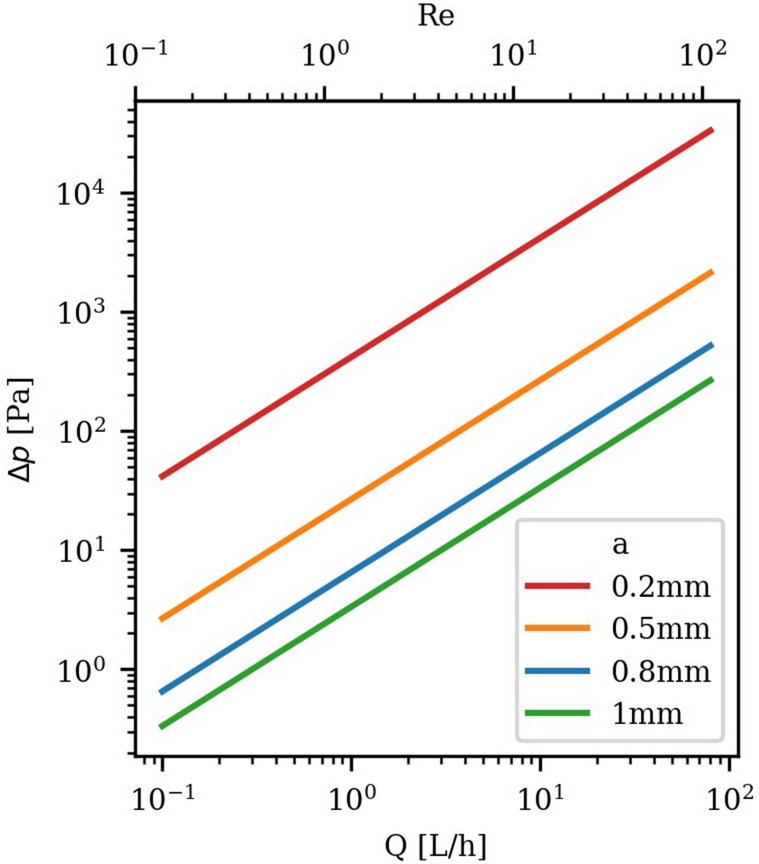
Design calculations for a laboratory fracture experiment: Pressure difference ∆P versus flow rate $\mathrm{Q}$ and Reynolds number $\mathrm{Re}$, calculated using the CL for a 20 cm × 20 cm parallel plate setting.

For the hydraulic testing, the required sensor range can be estimated by the following theoretical design calculations. For parallel plate-like experiments, $Re$ is defined as

1 $$\begin{array}{c}Re=\frac{\rho ua}{\mu }=\frac{Q\rho }{\mu w}\end{array}$$

where $u$ is the average flow velocity, $\rho$ the fluid density, $\mu$ the dynamic viscosity, $Q$ the volumetric flow rate, and $w$ the plate width. The $Re$ definition can be used to estimate the necessary $Q$ to range approximately from 0.072 L/h to 72 L/h for the desired $Re$ range ($0.1 < Re < 100$). To estimate the expected pressure difference $\Delta p$, we can use the analytic solution for laminar parallel plate flow for an incompressible fluid, known as the Cubic Law (CL)

2 $$\begin{array}{c}\Delta p=\frac{12 \mu L Q}{w {a}^{3}}\end{array}$$

where $L$ is the fracture length. Given that $a$ is much smaller than the fracture width ($a\ll w$), the parallel plate assumption is fulfilled by using planar plates. This Eq. (2) is used for the design calculations shown in Fig. 2. For $a=$ 1 mm and low $Q$ this results in low $\Delta p$, leading to the requirement of single-digit Pascal pressure measurements. In contrast, for low $a=0.2$ mm and high $Re$, $\Delta p$ of up to around 33 kPa need to be measurable. However, this calculation does not include nonlinear flow regimes which occur due to the fracture roughness at $Re \le$ 10^11^, leading to even higher $\Delta p$. Additionally, the system pressure ${p}_{sys}$ can be higher due to an outlet backpressure, resulting in the requirement of the pressure sensor measurement range up to the order of bars. The ${p}_{sys}$ at the pump position will be even larger due to the pressure losses in the pipes and additional control units before the fracture. Therefore, pumps need to handle high ${p}_{sys}$ of multiple bars as well as supplying $Q$ in the range calculated before. To prevent pumping induced flow disturbance, pump pulsation must be minimized. Given that many pumps exhibit ${p}_{sys}$ sensitive $Q$, additional $Q$ measurements are essential for verification.

Referring to the CL (Eq. 2), the mechanically most sensitive parameter is $a$. Experimentally, this leads to the need for a precise fracture positioning. The huge influence of $a$ on $\Delta p$ can be seen in Fig. 2, where the difference of 0.3 mm (from 0.2–0.5 mm at $Q=80$ L/h) results in a difference in $\Delta p$ of nearly 0.3 bar. Also the desired $a$ of less than 1 mm^36^ results in a necessary placement accuracy in the order of a few micrometers. Given that shear and its direction relative to the main flow direction significantly influences the fracture flow^20^ by affecting the channeling, the setup must permit controlled shear displacement across all directions within the fracture plane, with equally precise displacement control. Besides shearing, the flow channeling can also be caused by partially closed fractures, which may occur especially due to confining stresses at production wells, resulting in geometrically confined channels. Therefore, the case of partially closed fractures should also be realizable. To understand the occurrence and impact of channeling, local flow velocities need to be measured within the fracture.

From the setup design perspective, the fracture must be completely sealed along the edges even when sheared, to avoid bypass flow. The inlet and outlet should not interfere with the fracture flow. For undisturbed flow measurements, the inlet should provide a uniform inflow across the entire width, which can be supported by defining an entrance region or designing the inlet as a line source. The outlet must be designed to ensure a uniform back pressure distribution along the full width to avoid non-uniform flow paths due to the setup’s design. During experiments, complete fracture saturation is crucial. Trapped air would block parts of the fracture void, which would reduce the permeated volume and alter the flow paths directly influencing the overall fracture hydraulics.

While laboratory-scale experiments can be used for many unanswered questions about fracture flow, the upscaling to field-scale is another advanced challenge. While the self-affinity of the roughness simplifies the upscaling of the roughness structure over several orders of magnitude, Brown and Scholz showed that the scaling changes (particularly $H$) when various orders of magnitude are considered^17^. To test the influence of these changes in $H$, tests with artificial fractures of changed fracture roughness need to be capable. However, upscaling the fracture flow is even more problematic since the fluid properties, such as the viscosity, must also be scaled correctly to reach the same channeling effect, due to the aperture distribution, at laboratory-scale as at field-scale.

### **F**^**4**^**aT – Hydraulic Laboratory protocol**

**Fig. 3:**
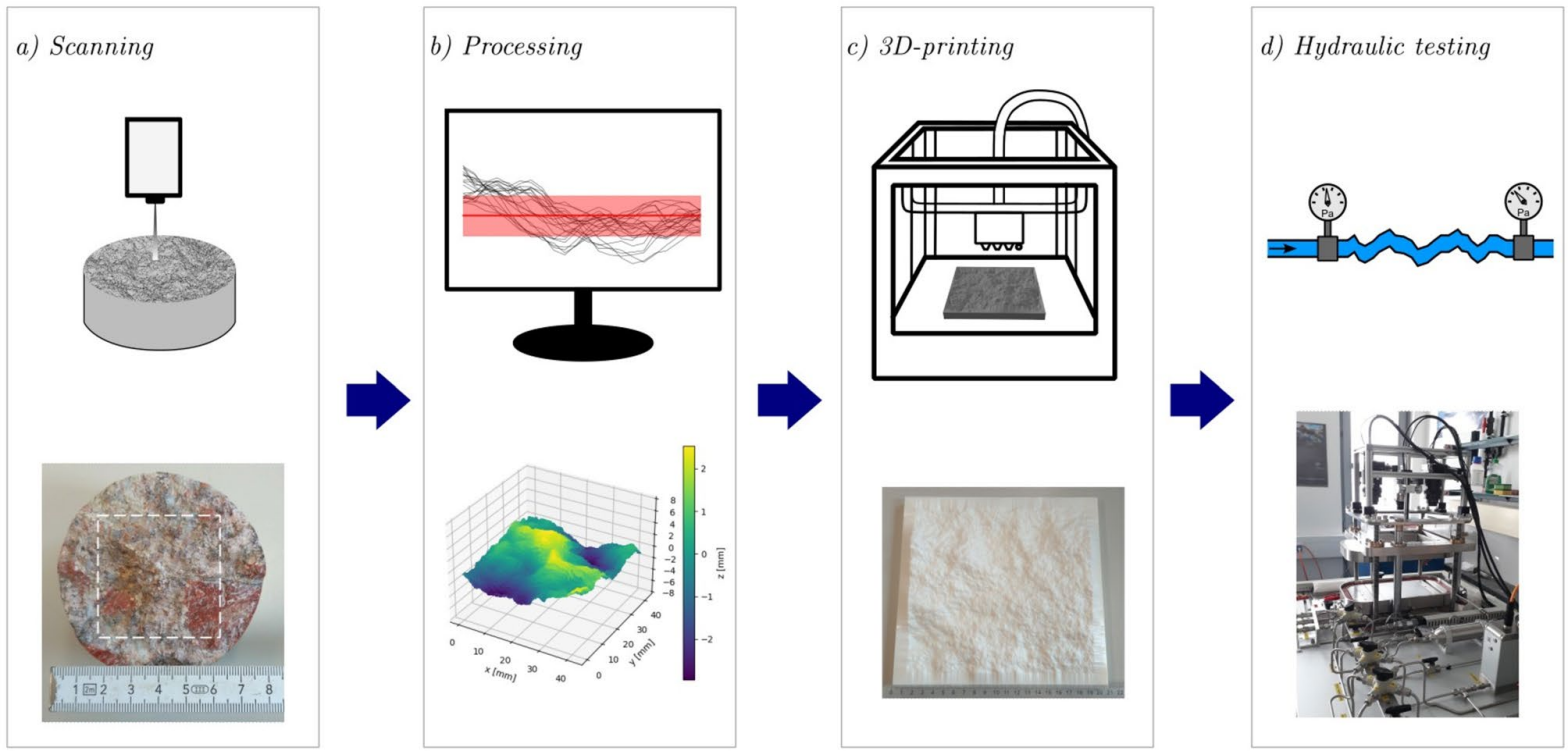
Complete experimental workflow of the F^4^aT-Hydraulic Laboratory: **a**) Scanning of natural rough rock surfaces with a 2D profilometer, **b**) analyzing the roughness statistics and creating a roughness replica, **c**) 3D-printing of the surface analog, **d**) characterizing the fracture hydraulics with the surface replica as boundaries in the F^4^aT flow-through test stand.

To experimentally investigate the relationship between roughness and fluid transport, the F^4^aT-Hydraulic Laboratory was established to fulfill the requirements outlined above. This laboratory was designed to study flow dynamics in well-defined rough fractures under systematic and controlled conditions. The full experimental protocol is visualized in Fig. 3 and described below. Further details of the measurements for each step are described in the Supplemental Information (SI 1).

The protocol starts by scanning rough rock fracture surfaces with an optical 2D profilometer (Mahr, MarSurf CP select) (Fig. 3a) to analyze the roughness of various fracture surfaces of different origin with a height resolution of 0.3 µm and a possible roughness measurement area to be over more than four orders of magnitude larger than the measurement resolution. These measured morphologies are processed (Fig. 3b) by analyzing roughness parameters, which can be utilized to create various fracture surface geometries, such as: (1) replicas of actual rock surfaces (direct copies of the scan), (2) basic geometric structures, such as double-sinus surfaces with the same $\sigma$, or (3) modified roughness profiles, for instance, extended or scaled versions, to systematically study the impact of specific roughness parameters on fracture flow. The created surfaces are extended to 3D models of the area 21 cm x 20 cm and a maximum roughness height of 2.8 cm. These models are 3D printed with a photopolymer resin at a resolution of 600 × 600 × 1600 dpi (Stratasys, Objet260 Connex1)(Fig. 3c) to create a defined transparent top and opaque bottom confinement for the fracture void geometry suitable for flow visualization, installed in the F^4^aT flow-through test stand (Fig. 3d). The two confining sides of the void are designed as identical, inverted rough models to be consistent with numerical studies^20^ and rock replication models^9,34,37^. Furthermore, the separate printing of these components also allows for the creation of fractures from two distinct opposing surfaces, for example to simulate highly sheared fractures. Slight shear of ± 2 mm and different apertures up to 1 cm can be set directly at the F^4^aT flow-through test stand, thereby allowing multiple fracture void geometries with the same printed surface. The printed surfaces have ramps at all edges to allow for shear and a uniform inflow over the entire fracture width. These fracture voids can be tested with a high range of $Q$ relevant for creeping fracture flow as well as for geothermal applications. Furthermore, the flow-through setup is built modularly, with the option to integrate additional systems like tracer experiments to quantify channeling, or a fluid temperature control unit to characterize fluid dynamics under varied fluid properties (see SI 2).

## Validation of hydraulic experimental setup

The flow-through setup was validated by the comparison of analytic CL calculations (Eq. 2) and parallel plate measurements, using smooth plates in the flow-through setup instead of rough rock replicas. In this configuration, the flow is known to remain laminar with linear hydraulic behavior at $Re$≤ 100 and can be solved analytically. The experimental series was initially split into two subsets, one with lower flow rates ($Q$ < 4 L/h) and one with higher flow rates ($Q$ > 4 L/h). This division was undertaken to first focus on the system’s calibration in laminar flow at low ${p}_{sys}$, before analyzing potentially deviating flow regimes.

**Fig. 4:**
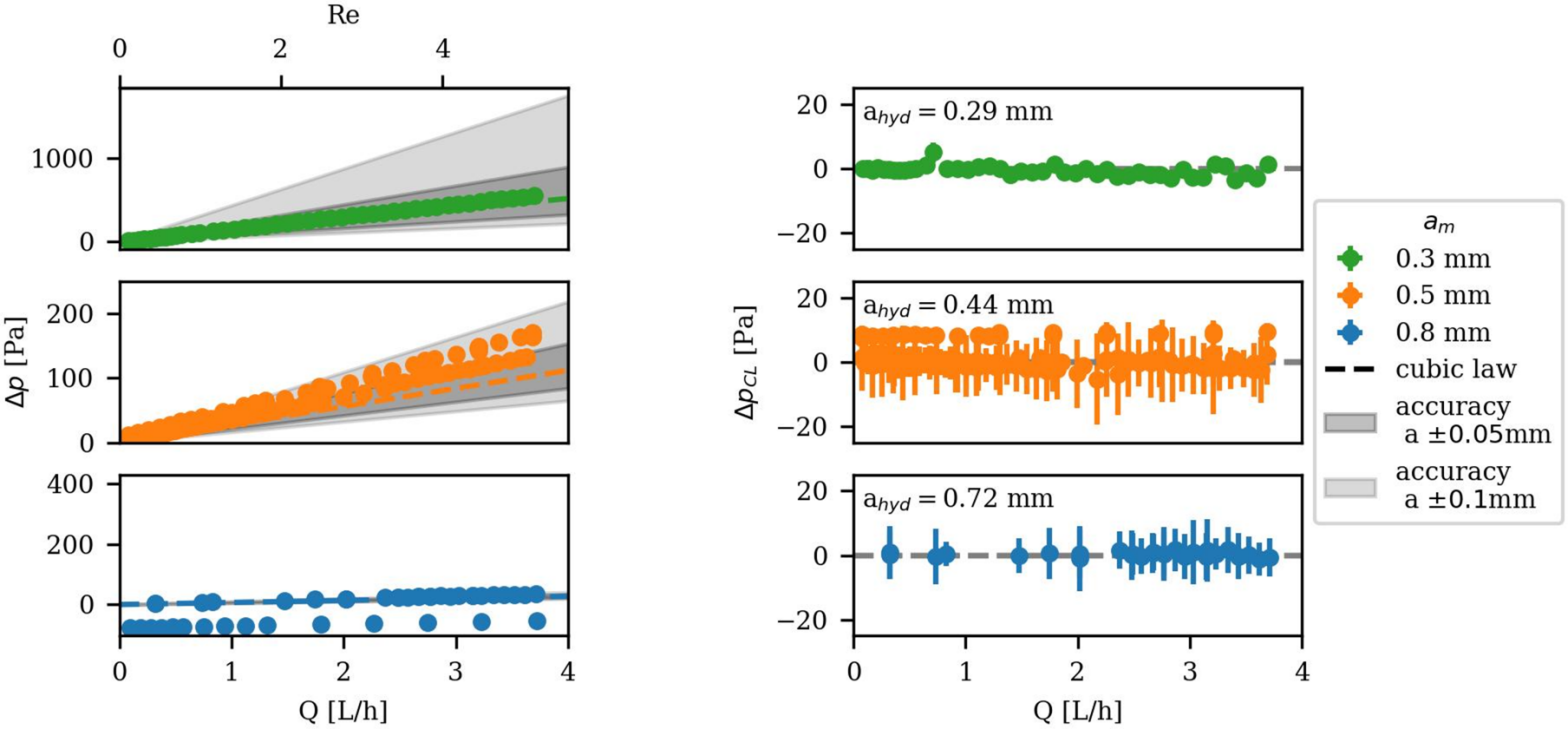
Hydraulic experimental measurement of the parallel plate setup compared with the CL theory for low flow rates: The total pressure difference ∆P over the flow rate $\mathrm{Q}$ and $\mathrm{Re}$ (left) with an aperture variation (accuracy) of ± 0.1 mm (bright shaded area) and for comparison a variation of ± 0.05 mm (dark shaded area). Additionally the reduced pressure difference $\Delta {\mathrm{p}}_{\mathrm{CL}}$ (right) to visualize the presission. For apertures of $0.3$ mm and $0.5$ mm one series of measurement are shown and for a = 0.5 mm 3 series of measurement. The three hydraulic apertures for $\mathrm{a}=$ $0.5$ mm are 0.454, 0.431, 0.424 mm.

For the low $Q$ measurements (Fig. 4), the hydraulic data $\Delta p, Q$ were recorded for $a=$ 0.5 mm three times. The fracture was reassembled in between each measurement to determine uncertainties in setting $a$. Additionally, $a=$ 0.3 mm and $a=$ 0.8 mm were measured. The total $\Delta p$ versus $Q$ and $Re$ is shown in Fig. 4 (left) to highlight measurement accuracy. The pressure error ${\sigma }_{p}$ is calculated with the propagation of uncertainty by ${\sigma }_{p}= \frac{\sqrt{\left({\sigma }_{in}^{2}+{\sigma }_{out}^{2}\right)}}{\sqrt{n}}$, where ${\sigma }_{in, out}$ is the standard deviation of the individual sensor measurements and $n$ the number of measured points. The measurement accuracy is primarily influenced by the aperture error, estimated to be 0.1 mm due to the mechanical mounting accuracy of the 3D printed fracture surfaces. The results show that all measurements are within the estimated aperture error range of ± 0.1 mm.

However, despite a slight deviation in slope due to the $a$ uncertainty, $\Delta p$ exhibit perfect linearity, indicating a precision in the order of just a few Pa, compared to the above defined measurement accuracy. To quantify the precision, the CL was fitted to low $Q$ data to determine the experimental hydraulic aperture ${a}_{hyd}=[\frac{12 \mu L{Q}_{measured}}{w\Delta {p}_{measured}}{]}^{1/3}$. The maximum deviation between this back-calculated ${a}_{hyd}$ and the targeted $a$ is 0.08 mm, lying within the estimated aperture error.

With the ${a}_{hyd}$, the theoretical hydraulic pressure difference $\Delta {\mathrm{p}}_{\mathrm{hyd}}$ was calculated via the linear CL (Eq. 2). Subtracting $\Delta {p}_{hyd}$ from the measured pressure difference $\Delta p$ results in the reduced pressure difference $\Delta {p}_{CL}=\Delta p-\Delta {p}_{hyd}$, which clearly demonstrates the linear hydraulic of the measurements, having a pressure precision of ± 2 Pa, as shown in Fig. 4 (right).

These results validate the flow-through setup at low $Q$ by showing the hydraulic linearity. The aperture uncertainty can be eliminated by the back-calculation of ${a=a}_{hyd}$, resulting in hydraulic measurements with a high precision in the single-digit Pascal range. Therefore, we continue this work using $\Delta {p}_{CL}$ for further analyses.

**Fig. 5:**
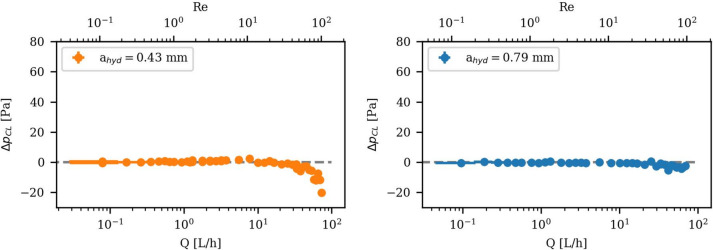
Hydraulic experimental measurement of the parallel plate setup for low to high flow rates: Pressure difference with subtracted the CL $$\Delta {\mathrm{p}}_{\mathrm{CL}}$$ in Pascal over the flow rate $$\mathrm{Q}$$ and $$\mathrm{Re}$$. The measurements are for two different apertures: $$\mathrm{a}=0.5$$ mm (left) and $$0.8$$ mm (right). The CL pressure differences at the highest $$\mathrm{Q}$$ are $$\Delta {\mathrm{p}}_{\mathrm{hyd}}(0.43$$ mm$$, 80$$ L/h$$) = 3495$$ Pa and $$\Delta {\mathrm{p}}_{\mathrm{hyd}}(0.79$$ mm$$, 80$$ L/h$$) = 537$$ Pa.

Extending the experimental range of $$Q$$ up to $$Re$$ = 100, $$a =$$ 0.5 mm and $$a=$$ 0.8 mm were measured. Figure 5 shows $$\Delta {p}_{CL}$$ for both cases. The results demonstrate good agreement with the CL up to medium $$Q$$ of around $$Re$$ **≈** 30. However, at $$a=$$ 0.5 mm a slight negative trend occurs in $$\Delta {p}_{CL}$$ at higher $$Q$$. This secondary effect is caused by minor fracture opening due to increased internal $${p}_{sys}$$. The highest measured $$Q$$ with $$\Delta {p}_{hyd}\left({a}_{hyd}= 0.43 mm, Q =80 L/h\right)= 3495 Pa$$, resulted in an increased $$a$$ of 1.2 µm, back-calculated from the ∆P deviation from the CL (Eq. 2). This effect is in absolute numbers more pronounced for the smaller aperture with around 20 Pa resulting in a relative ∆P underestimation of $$0.6\text{ \%}$$. For $$a=0.8$$ mm the ∆P deviation is around 5 Pa, being on the order of the pressure error, but resulting in a relative ∆P underestimation of $$0.9\text{ \%}$$ due to the lower $$\Delta {p}_{hyd}(0.8$$ mm$$, 80$$ L/h$$) = 537$$ Pa. Therefore, we conclude that the underestimation due to fracture opening is below $$1\text{ \%}$$ at our highest $$Q$$ measurements. At the specific $$Q$$ range of the onset of nonlinear hydraulic behavior of $$Re \le 10$$^11,30^, no fracture opening was observed.

## Investigation of rough fractures: proof of concept

In the following, the entire procedure of the F^4^aT-Hydraulic Laboratory protocol and its ability to show the influence of roughness on the hydraulics are demonstrated. As described above (Chapter 2.2), the protocol starts with the measurement and analysis of rock surfaces, here granite core surfaces, followed by the statistical reproduction of the sample replica, which is then used for the hydraulic measurements of $\Delta p$, $Q$.

### Analysis of surface morphologies

#### Natural rock surfaces

**Fig. 6:**
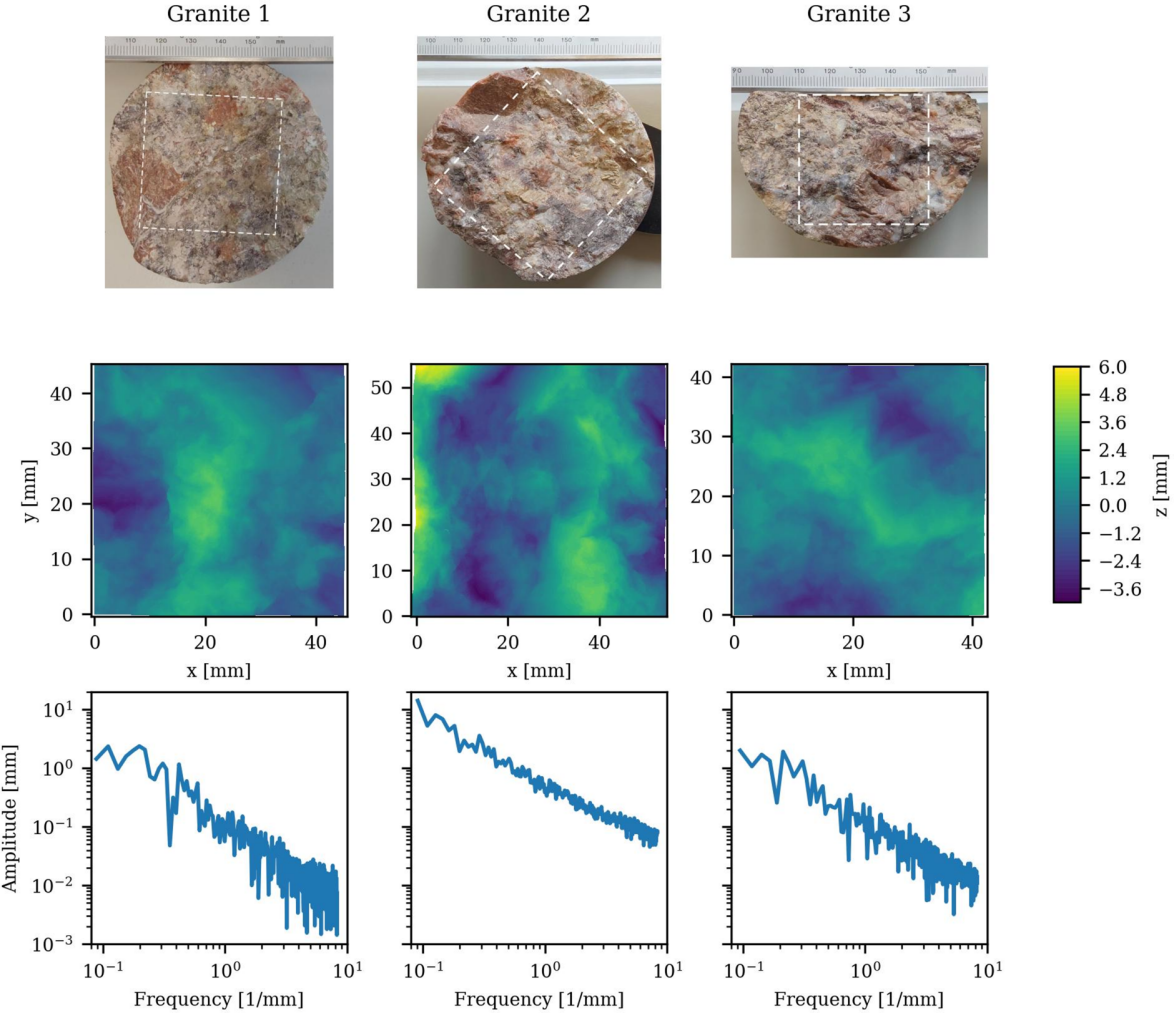
Pictures of the three analyzed granite core samples with indicated scanned area (top), the measured surface height profiles—overall horizontally-leveled (middle), and the frequency analysis of one representative line along x (bottom).

Three granite samples from cored geothermal wells from Soultz-sous-Forêts, France, having macroscopically fresh fracture surfaces with no signs of alteration, were analyzed to characterize their surface roughness. The three granite samples are presented in Fig. 6 (top). The scan areas are marked by white dashed squares. The height profiles (Fig. 6 middle) of the sizes declared in Table 1 were scanned with a horizontal resolution of 30 µm × 30 µm, matching the printing resolution of the roughness replica. The roughness frequency was analyzed to verify the roughness self-affinity, which requires a linear trend in log–log representation. For illustration purposes, one representative line for each sample is plotted in Fig. 6 (bottom) showing the occurrence of roughness across all measured frequencies with a linearly increasing roughness amplitude at lower frequencies. The linear relationship, observed for all samples, confirms a universal self-affine surface roughness at these scales. Possibly due to the larger scanned area, granite 2 displays larger amplitudes across all frequencies, while granite 1 and 3 exhibit lower, relatively similar roughness amplitudes. All three samples have comparable amplitude slopes, indicating comparable $H$ values.

**Table 1:** Surface analysis for the scanned granite surfaces and the roughness replica: the maximal area after rotation, the original scan resolution, the mean Hurst exponent $H$, the maximum height difference Δz_tot_, the standard deviation of the height ${\boldsymbol{\sigma}}$(z_tot_) and the sub-window standard deviation ${\boldsymbol{\sigma}}$(z_10mm_^2^).

Surface	Area [mm]	Resolution [µm]	H [-]	Δz_tot_ [mm]	${\boldsymbol{\sigma}}$(z_tot_) [mm]	${\boldsymbol{\sigma}}$(z_10mm_^2^) [mm]
Granite 1	45.5 × 45.6	30	0.86 ± 0.05	7.15	1.31	0.32 ± 0.16
Granite 2	56.1 × 55.1	30	0.84 ± 0.07	10.01	1.74	0.40 ± 0.12
Granite 3	42.4 × 42.4	30	0.87 ± 0.05	5.81	1.26	0.30 ± 0.13
Replica	200 × 200	100	0.83 ± 0.04	21.13	3.43	0.32 ± 0.12

In Table 1, the detailed analyzed values of the three measured surfaces regarding the scanned area, the scanning resolution, and the analyzed roughness parameters are specified. The given area is defined as the maximum 2D area after horizontal leveling by orienting the average surface plane horizontally. $H$ is calculated by the 1D R/S-analysis^38^, also referred to as rescaled range. There, H can be extracted via a fit of $\frac{R(n)}{S(n)}=C* {n}^{H}$ from the $R/S$ data over $n$, with $n$ being the number of data points of a window part of the full line, $S(n)$ is $\sigma$ of this window, $R(n)$ the maximal difference of the cumulative sum of the height deviation from the mean value within the measurement window and $C$ a constant. Multiple 1D lines, spaced parallel 3 mm apart, were analyzed for 34 equally spaced direction angles to determine the average $H$. Fault roughness measurements by Candela^16^ resulted in anisotropic $H$ varying by 0.23. In comparison with Candela’s results, our measured variations in $H$ of a maximum of 0.07 argue for isotropic roughness of all samples, resulting in the assumption of the samples representing joint roughness. Although these joints can be found in conventional hydrothermal systems and groundwater flow, they are not promising for EGS due to their likely confining stress-caused complete closing after stimulation. Their occurrence on the Soultz-sous-Forêts site brings up the question whether their origin is drilling induced. A further stress field analysis in comparison with borehole log-data would go beyond the scope of this paper, but will need to be considered in further research.

To account for the roughness amplitude (scale of z-extension), we additionally provide the maximum sample height differences Δz_tot_ and the total height standard deviations $\sigma ({z}_{tot})$ in Table 1. Granite 2 shows a significantly higher maximum height difference than the other samples, consistent with Fig. 6. Since Δz_tot_ and $\sigma ({z}_{tot})$ of self-affine rough surfaces are highly dependent on the scan area, the standard deviation of a of 10 mm^2^ sub-area ${\boldsymbol{\sigma}}$(z_10mm_^2^) was analyzed. A moving window approach was used to cover the entire measured surface to reduce the influence of local variations. This analysis demonstrates comparable local roughness amplitudes across all three surfaces, while minimizing the effect of the different scan areas.

However, this roughness analysis can just be seen as a representative example due to the neglected analysis of their origin and the associated stresses. Additionally, just 3 samples were analyzed, even with their highly comparable roughness, their amount is barely sufficient for comparing statistics of the samples, with altered fracture surfaces, or with samples from different sites.

#### Replica characterization

A roughness replica is used in the flow through setup to enable experiments with defined fractures with the option to vary single roughness parameter. In this case, the roughness replica was designed by maintaining statistical similarity in roughness characteristics to those of the measured rock surfaces. The method described below was used to create the replica, allowing to accommodate the constraints of the flow-through setup and statistically extend the measured roughness.

**Fig. 7:**
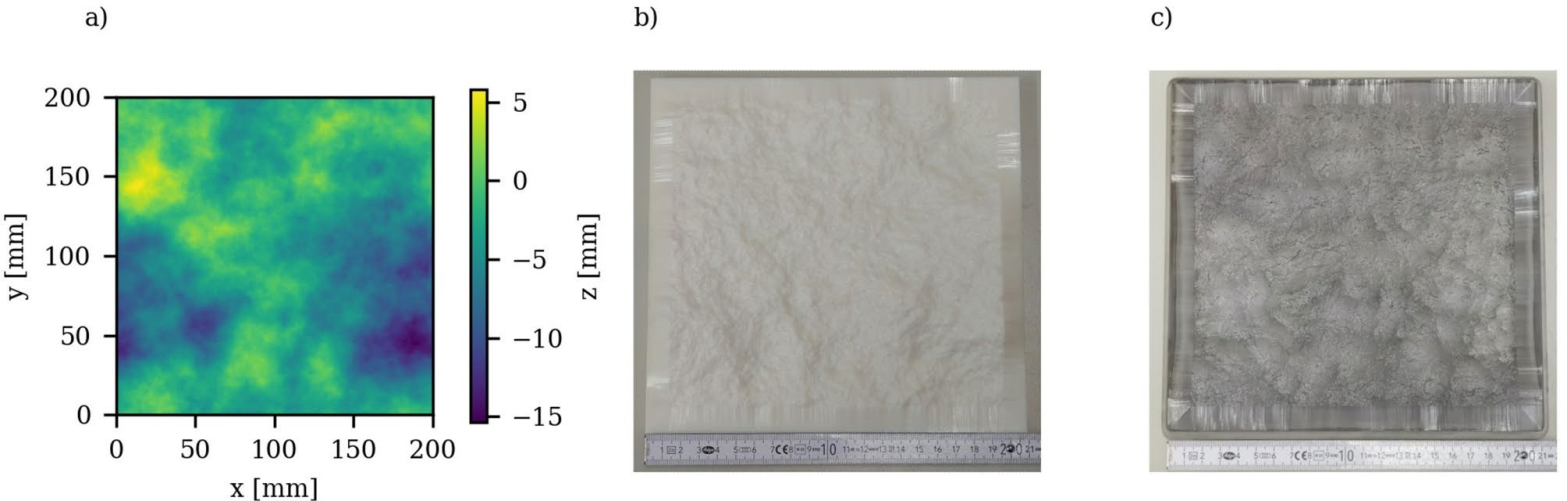
Roughness replica: Roughness height map of the created replica roughness (**a**) and pictures of 3D-printed fracture surfaces: white bottom (**b**) and transparent top (**c**).

The SAFFT software, developed by Schmittbuhl^39^, was employed for generating the roughness replica. In this process, slight variations in $H$ were considered, applying an isotropic $H = 0.8$, which is typical for granite, across a grid consisting of 2048 × 2048 nodes. This generated surface was scaled to the dimension of 20 cm in x and y direction to conform to the specifications of the flow-through setup and to consider in-/outflow ramps of at least 5 mm each. The replica’s height was scaled using ${\boldsymbol{\sigma}}$(z_10mm_^2^) of granite 1. The resultant height profile is displayed Fig. 7(a). To control the generated surface, the same roughness analysis was performed as on the natural samples, with the results summarized in Table 1. The replica roughness’s $H$ is consistent with those observed in the natural surfaces. Owing to the larger size of the self-affine replica surface, its $\Delta {z}_{tot}$ is in the order of centimeters larger. However, the subarea analysis of ${\boldsymbol{\sigma}}$(z_10mm_^2^) remains consistent with that of granite 1, and even the height variations within these subareas remain comparable to those of the measured samples. This surface was used to create 3D models featuring inverted, matching rough surfaces to enable complete fracture closure. Ramps were incorporated at all replica margins. Figure 7(b, c) displays images of the printed surface structures, which are devoid of visible printing artifacts.

### Hydraulic results of the rough surface replica

**Fig. 8:**
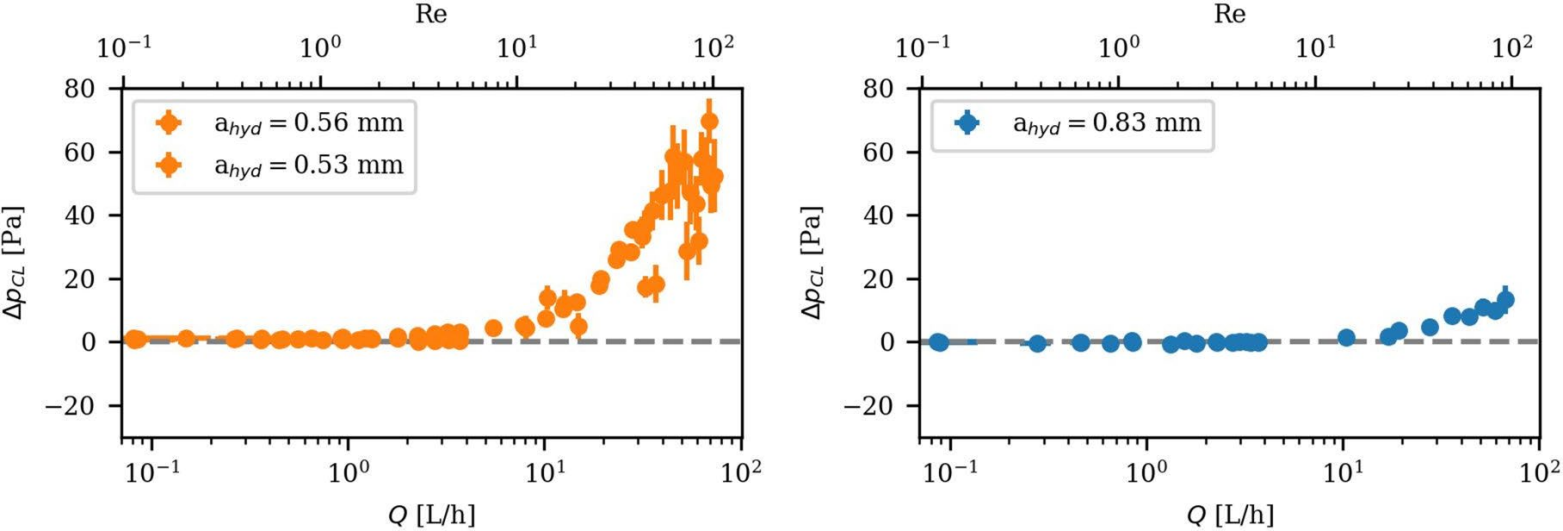
Hydraulic experimental measurement of a rough fracture for low to high flow rates: Reduced pressure difference with subtracted CL $\Delta {\mathrm{p}}_{\mathrm{CL}}$ in Pascal over the flow rate $\mathrm{Q}$ and $\mathrm{Re}$.

The printed fracture replica was used in the F^4^aT flow-through setup to measure $\Delta p$ at the full $Q$ range of the rough fracture with vertical $a$ of 0.5 mm and 0.8 mm, conforming with the mean local apertures of 518 µm and 748 µm for unaltered granite measured by Sausse^36^. Since the sample roughness showed no significant anisotropy (Chapter 4.1.1), the roughness was assumed to reflect a rock joint, so the experiment was set up with a normally opened geometry. The measured hydraulic results for the two different $a$ are shown in Fig. 8. To highlight deviations from the linear CL, $\Delta {p}_{CL}$ is presented. For rough surfaces, it should be noted here that setting $a = {a}_{hyd}$ does not just correct the mechanical aperture. Furthermore, it already includes the average effect of tortuosity over the entire fracture, neglecting increases in tortuosity caused by non-uniform flow patterns. Therefore, this method cannot be applied for questions regarding e.g. the tortuosity dependence on shear and aperture, but is suitable for an overall fracture hydraulic investigation.

At low to moderate $Q$, $\Delta p$ shows a linear relation to $Q$, fully consistent with the CL. However, starting at approximately $Re$ ≤ 10, the hydraulic behavior deviates from the CL approximation with increasing Q, exhibiting disproportionately higher $\Delta p$. This result is in good agreement with previous experimental studies by Zimmerman^30^ and numerical calculations by Egert^11^, showing the onset of nonlinearity below $Re$= 10.

The relative $\Delta {p}_{CL}$ should be higher at lower $a$ due to the higher relative roughness^13,33,40^, defined by the ratio of roughness height divided by $a$. The presented measurements indicate the same result, but with an uncertainty due to the measurement error and possible fracture opening (see chapter 3). The maximal measured $\Delta {p}_{CL}$ normalized by CL at the same value for $Q$ is around 3% for $a =$ 0.5 mm and 2.5% for $a =$ 0.8 mm. Additional measurements with different $a$ are needed to validate the relation. The onset of the hydraulic non-linearity occurs long before the onset of the fracture opening. For a quantitative analysis of the non-linear deviation regarding $a$, shear, and fracture roughness, the fracture aperture needs to be measured with higher sub-micrometer resolution and subsequently corrected. Alternatively, the measured onset of fracture opening needs to be taken into account and flow rates just up to that onset should be considered. By including one of these steps, the fraction of non-linearity depending on a, H, std can be evaluated.

## Conclusion

Based on the validation of the setup with parallel plates, the first rough fracture experiments utilize the F^4^aT-Hydraulic Laboratory to investigate flow in rough rock fractures at a large $Q$ range relevant for creeping groundwater fracture flow as well as for high flow rate geothermal energy production with $Re$ ranging from $Re$ « 1 to 100. The approach includes measuring and analyzing natural rough rock fractures, producing an accurate roughness replica, and executing hydraulic tests on the replicated rough surface. With a back-calculated $a$ correction, the $\Delta p$ measurements reached a precision of $\pm 2$ Pa. Furthermore, at high $\Delta {p}_{sys}$ due to the high $Q,$ the setup remained stable with minor movement, in the order of a micrometer, leading to $\Delta p$ deviations below 1%, only appearing at high $Q$ ($Re$ ≥ 30). This precision of $\pm 2$ Pa at a large $Re$ range supports precise, reproducible experiments and validations of numerical models.

The capability of measuring non-linear hydraulic effects due to the fracture roughness was demonstrated based on granite samples from the cored wells of the Soultz-sous-Forêts geothermal production site in France. The hydraulic measurements of the rough, replicated fractures showed the transition from the linear to nonlinear flow behavior, initiating at $Re$ ≤ 10, by showing significant over-proportional $\Delta p$ deviations at high $Q$. These outcomes are consistent with existing literature^11,30^, supporting this approach for future stochastic investigations.

The F^4^aT-Hydraulic Laboratory facilitates a systematic and stochastic examination of the interplay between rock fracture surface roughness and hydraulic fracture properties. This endeavor includes precise roughness characterization and diverse hydraulic testing of systematically varying fracture roughness, extending it to sheared fracture configurations and tracer studies, thereby complementing numerical simulations.

## Supplementary Information

Supplementary Information.

## Data Availability

Data sets generated during the current study are available from the corresponding author upon reasonable request.
