# Supplementary material for: Implementation and validation of the F4aT laboratory for flow in rough fractures
Source: Sci Rep. 2026 Jan 13;16:1956. doi: 10.1038/s41598-025-34648-2 (PMC12804876; doi:10.1038/s41598-025-34648-2)
Supplement: Supplementary file 1 — Supplementary Information. [file 41598_2025_34648_MOESM1_ESM.docx]

Supplemental information

# Details of the F^4^aT-Laboratory

The following sections provide supplementary details regarding the experimental capabilities and components of the F^4^aT Laboratory, expanding upon the methods section of the manuscript.

## Roughness scanning

The optical 2D profilometer (Mahr, MarSurf CP select) is equipped with a chromatic sensor (CLA 10). This system provides a horizontal resolution down to 5 µm × 5 µm and a height resolution of 0.3 µm, with a maximum scanning area of 20 cm × 20 cm. This enables the roughness measurement area to be over more than four orders of magnitude larger than the measurement resolution. The sample surfaces are manually oriented horizontally for the scanning to minimize the risk of overhangs in the sample roughness, which are unmeasurable by the profilometer from vertically above. The scan area can be chosen freely as a rectangular area to avoid measuring possible edge break-offs.

## Roughness analysis and processing

Post-scanning, the scanned surface can be post-processed with arbitrary analyses. One main important step before most of the analysis is the leveling of the measured surface by orienting the average surface plane horizontally for consistency. From this leveled surface, roughness parameters such as the in the manuscript calculated $H$ and $\sigma$ are determined.

## Artificial rock replica and printing

In the flow-through test stand, fracture models with a horizontal dimension of 21 cm × 20 cm, and a maximum possible roughness height of 2.8 cm can be installed. The hydraulic connection between the rough fracture and the setup is a critical point, which is designed as a line source and sink for the inlet and outlet of the fracture, meaning the rough fracture models need ramps at the surface inlet and outlet edges, assuming a minimal length of 5 mm each, to enable water inflow and outflow across the full fracture width. Additional ramps at the sides of the surfaces enable fault experiments with a setup shear component perpendicular to the main flow direction. These ramps are designed with a length that ensures gradients lower than the steepest replica surface slope over a distance up to 2 mm, which is the maximum shear distance permitted by the flow-through setup. This ensures that $a$ is controlled by the roughness of the replica surface **instead of** the slope of the ramps during shearing, assuming the fracture void model with one contact point.

The generated 3D models with the defined roughness for the fracture top and bottom are printed separately using a 3D printer (Stratasys, Objet260 Connex1) at a resolution of
600 x 600 x 1600 dpi. The bottom surface is printed with solid white photopolymer resin (alphacam, VeroPurewhite), while the top surface is printed with transparent photopolymer resin (VeroClear^TM^). This enables the visual verification of the fracture saturation as well as the monitoring of future tracer transport experiments within the fracture.

## Hydraulic experiments

For hydraulic measurements, the printed surfaces are mounted as the upper and lower confinement of the fracture void within the novel flow-through setup (SolExperts AG). The fracture is sealed with a surrounding inflatable rubber seal and additionally with a 5 mm wide sealing clamped at the sides, narrowing the fracture width down to 19 cm. Precise positioning sensors (Heidenhain, SPECTO ST1288) are employed to control the fracture's position and $a$, with the maximum $a$ reaching 1 cm and the maximum shear in both horizontal directions being ±2 mm, which can also be combined for possible shear in any horizontal directions. The fracture surface’s position is monitored with 1 µm accuracy in all spatial directions. The overall mechanical $a$ accuracy, considering the mounting uncertainty is estimated to be 0.1 mm.

For the hydraulic measurements, especially the design of the inlet and outlet are of special relevance. The inlet is designed as a uniform line source, where the flow from a pipe is expanded to the full width by a comb finger structure. To additionally reduce pressure distortions from the inlet and outlet which would affect the fracture flow, 3 cm of parallel plates are located upstream and downstream the fracture with the same $a$ as the fracture. A reservoir before the outlet pipe maintains uniform pressure distribution downstream of the fracture over the full width. The outflow is directed into an overflow approximately 20 cm above the fracture to achieve a slight, constant back pressure to preserve the full fracture saturation even at low $Q$.

The flow is controlled by two gear pumps: one for low $Q$ (HNP Mikrosysteme, mzr-4665) and another for high $Q$ (GATHER Industrie, LAB 12), supporting together $Q$ ranges between
0.08-80L/h. Minor $Q$ fluctuations from the pumping are cushioned by a partially air-filled reservoir upstream the fracture inlet. $Q$is monitored using two flowmeters (Bronkhorst, mini CORI-FLOW M13/M15) with rated accuracies of 0.2%Rd+ 50g/h (M15) and 0.2%Rd+ 0.2g/h (M13). The fracture pressure is recorded by membrane pressure sensors (Keller, Serie-33X), with 5 Pa total error, at hydraulic dead ends centered behind the inlet and outlet. To enhance pressure accuracy, and mitigate room-air and system movement effects, $\Delta p$ of each $Q$ are recorded over several minutes at a rate of 2/s, once $\Delta p$ and $Q$ stabilized.

The hydraulic measurements are performed using deionized water, which can be also replaced by other working liquids, and the full saturation is visually confirmed through the transparent top surface.

# Future experiments

The modularity and capabilities of the F^4^aT flow-through setup allow for the investigation of complex, advanced flow scenarios crucial for understanding fractured reservoirs. The following subsections outline two important future experimental settings.

## Tracer experiments

The fracture roughness leads to variations in the local fracture aperture, which become more pronounced with increasing fracture shear. These aperture variations influence fracture flow, resulting in channeling effects where the primary flow is concentrated in high-permeability paths and is not uniformly distributed. To quantify this channeling effect, direct measurements within the fracture are required. With tracer experiments, these channels can be visualized in the F4aT flow-through setup by recording through the transparent top fracture. A valve upstream of the inlet directs the fluid through an additional tracer line. This configuration allows for the analysis of channeling as a function of flow rate (investigating transport processes across different hydraulic regimes) and various fracture geometrical properties, such as sheared or partly closed configurations. In addition to analyzing the tracer distribution over time, the movement of the tracer front will indicate local flow velocities, which are critical for developing a detailed fluid dynamic understanding within the fracture. For comparison with field data, tracer flow-through curves can be generated by integrating the tracer intensity over the fracture width or by measuring the intensity in the pipe downstream of the fracture outlet, providing insight into the impact of channeling on overall transport processes.

Obtaining more detailed data on local flow velocities requires more advanced measurement techniques like particle image velocimetry or particle tracking velocimetry. The transparent top facilitates the implementation of these techniques. However, the higher resolution requires more rigorous planning, particularly concerning refractive index matching, tracer particle selection, and the appropriate choice of camera resolution and frame rate. Consequently, the implementation of these advanced techniques remains a subject of future work.

## Viscosity dependency

In Enhanced Geothermal Systems (EGS), the viscosity of brine differs significantly from that of water used at room temperature, owing to elevated temperature and salinity. The setup can be combined with a heating tank, allowing the working liquid temperature to be controlled from room temperature up to 40 °C, which is the maximum operational limit for the currently used polymer resin fractures. Temperature sensors at the fracture inlet and outlet will verify temperature stability across the fracture or record any temperature gradient if the system has not reached thermal equilibrium with the fluid.

While these temperatures are low compared to typical geothermal applications, the pump and flow meter combination permits the use of alternative liquids or mixtures to achieve a more drastic viscosity change. For viscosity reduction, liquids such as ethanol, methanol, or acetone could be employed. Conversely, a glycerin-water mixture could be used to increase viscosity and enhance temperature sensitivity. This flexibility allows for viscosity variations without requiring a complete fluid exchange between experiments. The option of a closed-loop system, where the working fluid circulates, significantly reduces the required fluid volume.
